# Cardiac magnetic resonance T2 mapping for monitoring acute cardiac transplant rejection

**DOI:** 10.1186/1532-429X-13-S1-P341

**Published:** 2011-02-02

**Authors:** Asad A Usman, Kirsi Taimen, Marie Wasielewski, Saurabh Shah, Jermey D Collins, Jennifer M McDonald, James C Carr

**Affiliations:** 1 Northwestern University, Chicago, IL, USA; 2 Siemens Medical Solutions, Chicago, IL, USA

## Objective

To assess the utility of cardiovascular magnetic resonance (CMR) in acute cardiac rejection using T2 mapping.

## Introduction

Cardiac transplantation is the treatment for some patients with end-stage heart failure. After transplantation asymptomatic acute allograft rejection is a major factor impacting survival in the first 12 months. Current transplant monitoring requires frequent right heart catheterizations, endomyocardial biopsies (EMB), and echocardiography. CMR imaging, comparatively less invasive, has been studied previously in the transplanted heart and prolonged T2 relaxation has shown correlation to transplant edema and rejection [1]. We hypothesize that prolonged T2 relaxation in transplant edema reflects rejection, and that quantitative T2 mapping will correlate with pathological and clinical findings.

## Methods

Patients were recruited from the transplant clinic for CMR within the first year of transplantation or if admitted to hospital for rejection. All MRI scans were performed within 24 hours of EMB. Biopsies were graded according to the International Society for Heart Lung Transplant grading system for cellular rejection with or without immunohistochemistry (IF) marking humoral rejection.

We used a non-contrast multiplanar single-shot and cine TrueFISP imaging sequence. Each patient also underwent a novel four-chamber and three short axis quantitative T2 mapping sequence using a single-shot T2-prepared SSFP acquisition with three T2-prep echo times: 0, 24, and 55 msec. T2 maps were analyzed using the AHA 17 segment model independently by two reviewers (AU and MW) blinded to outcomes for inter-rater and intra-rater reliability.

## Results

**Figure 1:**
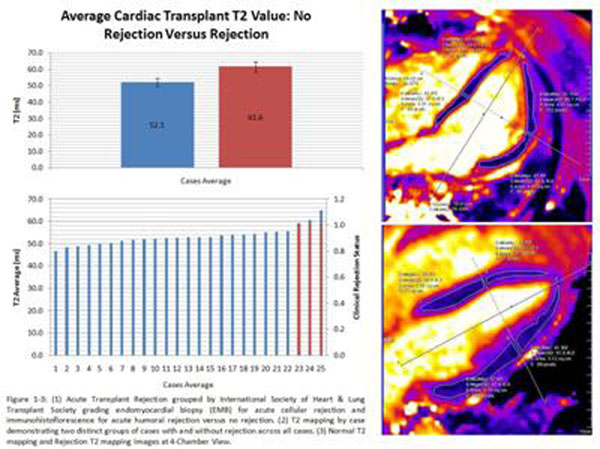


A control cohort of 20 cases demonstrated a normal T2 average of 51.2 ± 2.7 ms. A total of 25 transplant scans were performed with average age 55.8 ± 13.8. All patients were on immunosuppressant regimen of prednisone, tacrolimus, with or without mycophenolate mofetil. There were two cellular and one humoral rejections. The average T2 relaxation time in patients with 0R/1R and negative IF was 52.1 ± 2.4 ms versus 61.6 ± 3.1 ms in rejections (p<0.05). The average ejection fraction for the rejection versus non-rejection cases was not significantly different, 58.7 ± 12.7% versus 53.1 ± 2.2% (p>0.05). All rejection cases were rescanned and demonstrated T2 value resolution with treatment. Figure 1

## Conclusion

Preliminary results demonstrate that T2 mapping offers a novel non-invasive tool for transplant monitoring for both cellular and humoral rejection. A larger multi-institution study will help elucidate the sensitivity and specificity of T2 mapping and the possibility of becoming an adjunctive tool in routine transplant monitoring.
